# Chronic Endometritis and Endometrial Polyps in Female Infertility: Etiological Mechanisms, Diagnostic Insights, and Prognostic Implications

**DOI:** 10.3390/jcm15187045

**Published:** 2026-09-11

**Authors:** Zofia Maria Kiestrzyn, Tatiana Antczak, Jakub Dyś, Maciej Wilczak, Karolina Chmaj-Wierzchowska

**Affiliations:** 1 Faculty of Medicine, Poznan University of Medical Sciences, 60-701 Poznan, Poland; tati.antczak@gmail.com (T.A.); kbudsy@gmail.com (J.D.); 2 Student Scientific Society of Obstetrics and Gynecology, Poznan University of Medical Sciences, 10 Fredry Street, 61-701 Poznan, Poland; 3 Department of Maternal and Child Health and Minimally Invasive Gynecologic Surgery, Poznan University of Medical Sciences, 60-701 Poznan, Poland; mwil@ump.edu.pl; 4 Laboratory of Advanced Interventional Therapies in Gynecology and Urogynecology, Poznan University of Medical Sciences, 60-701 Poznan, Poland

**Keywords:** chronic endometritis, endometrial polyps, infertility, CD138, hysteroscopy, endometrial microbiota, polyp recurrence, endometrial receptivity

## Abstract

**Background/Objectives:** Endometrial polyps (EPs) are common benign intrauterine lesions associated with abnormal uterine bleeding, infertility, recurrent implantation failure, and recurrent pregnancy loss. Although traditionally considered hormone-dependent lesions, increasing evidence suggests that chronic endometritis (CE) may contribute to their development and recurrence. This review summarizes current evidence regarding the relationship between CE and EPs, focusing on pathophysiological mechanisms, diagnostic approaches, and clinical implications. **Methods:** This narrative review was conducted according to the Scale for the Assessment of Narrative Review Articles (SANRA). A literature search was performed using PubMed/MEDLINE, Scopus, and Google Scholar to identify original studies, systematic reviews, meta-analyses, and consensus statements addressing CE, EPs, infertility, endometrial microbiota, and polyp recurrence. **Results:** Current evidence demonstrates a consistent association between CE and EPs, with approximately half of premenopausal women with EPs showing histological evidence of CE. Chronic inflammation may promote polyp formation through immune activation, inflammatory cytokine signaling, abnormal angiogenesis, extracellular matrix remodeling, dysregulated apoptosis, and alterations in the endometrial microbiome. CE has also been identified as an independent predictor of EP recurrence after hysteroscopic polypectomy and may impair endometrial receptivity. Histopathological examination with CD138 immunohistochemistry remains the diagnostic reference standard. **Conclusions:** Emerging evidence supports an inflammatory component in the pathogenesis of endometrial polyps and suggests that CE may represent a potentially modifiable risk factor for recurrence and reproductive failure. Incorporating assessment of CE into the evaluation of selected women with recurrent polyps or infertility may improve individualized management. However, prospective studies are needed before routine screening and treatment can be universally recommended.

## 1. Introduction

### 1.1. Endometrial Polyps

**Figure 1:**
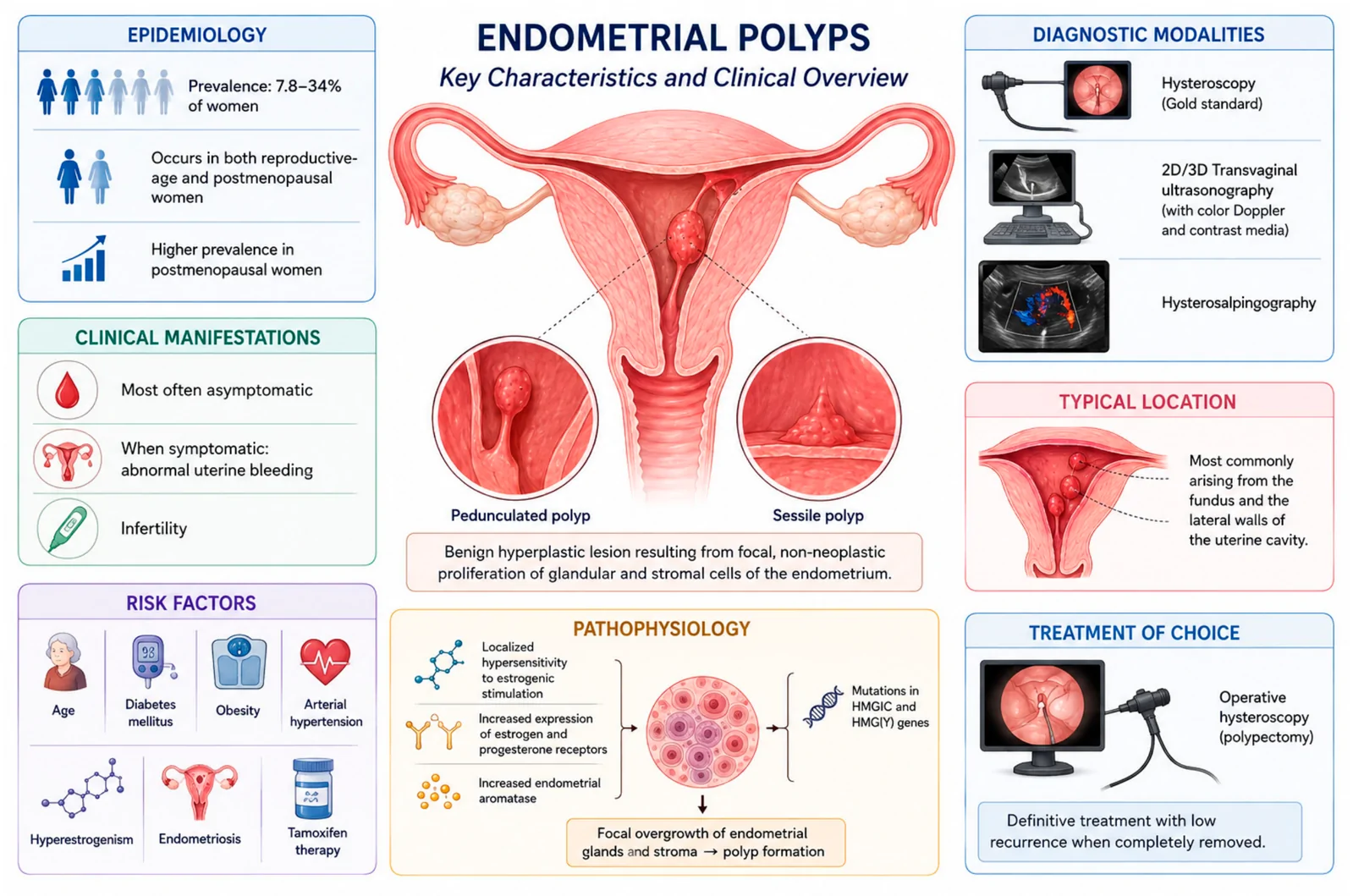
Endometrial polyps: Epidemiology, pathophysiology, clinical presentation, diagnosis, and treatment.

An endometrial polyp (EP) is defined as a benign hyperplastic lesion resulting from the focal, non-neoplastic proliferation of the glandular and stromal cells lining the uterine cavity. These lesions can present in diverse morphological forms, ranging from pedunculated structures moving freely within the uterine cavity to sessile lesions with a broad base attached to the myometrium. Endometrial polyps affect an estimated 7.8% to as many as 34% of women [1]. They occur in both reproductive-age and postmenopausal women, with a higher prevalence observed in the latter group. Diagnostic modalities for endometrial polyps include hysteroscopy, two- and three-dimensional transvaginal ultrasonography—particularly with the application of color Doppler and contrast media–as well as hysterosalpingography [1,2]. Risk factors for polyp formation include age, diabetes mellitus, obesity, arterial hypertension, hyperestrogenism, endometriosis, and tamoxifen therapy [1,2,3]. The pathophysiology of polyp development is complex, yet it is classically associated with a localized hypersensitivity to estrogenic stimulation; these lesions are not observed prior to menarche, and an increased expression of estrogen and progesterone receptors, endometrial aromatase, as well as mutations in the *HMGIC* and *HMGI(Y)* genes are emphasized in the formation pathophysiology [1,4]. Endometrial polyps are frequently asymptomatic lesions, which partly accounts for the wide discrepancy in estimates of their prevalence. However, when they become symptomatic, the most common clinical manifestations are abnormal uterine bleeding and infertility [1,5,6,7,8,9]. The preferred method of treatment for endometrial polyps is operative hysteroscopy [1,5,10]. An overview of the epidemiology, pathophysiology, clinical presentation, diagnostic work-up, and current management of endometrial polyps is presented in Figure 1, providing the clinical background for the subsequent discussion on the role of chronic endometritis in endometrial polyp development.

### 1.2. Chronic Endometritis

Chronic endometritis (CE) is a persistent, localized inflammation of the endometrial lining, characterized by plasmacytic infiltration within the stroma. In contrast to acute endometritis, which presents with a severe clinical course, CE is predominantly asymptomatic or oligosymptomatic. Due to the absence of specific symptoms (such as mild pelvic pain, abnormal uterine bleeding, or vaginal discharge), this condition frequently remains undiagnosed [1,10,11,12].

### 1.3. Chronic Endometritis (CE) as a Risk Factor for Endometrial Polyp Formation

In recent years, a growing body of evidence in the medical literature has indicated an association between chronic endometritis (CE) and the development of endometrial polyps (EPs). Although polyps—which are benign, localized overgrowths of endometrial glands and stroma—have traditionally been attributed primarily to hormonal imbalances (e.g., hyperestrogenism), it is currently postulated that the inflammatory pathway plays an equally significant role in their pathophysiological association [13,14,15]. It has been demonstrated that in patients with endometrial polyps, the prevalence of CE reaches 51% [13]. Additionally, the prevalence of CE is twofold higher in the presence of EPs compared to their absence [14]. Furthermore, a strong correlation (*p* < 0.0001) has been established between the presence of micropolyps and the histopathological diagnosis of CE utilizing the CD138 marker [15]. From the perspective of gynecological practice and reproductive medicine, undiagnosed chronic endometritis is considered a significant associated factor of recurrent polyps. Furthermore, persistent endometrial inflammation following surgical excision has been associated with an increased risk of polyp recurrence in observational cohorts [13,15], highlighting the importance of evaluating background tissue characteristics.

### 1.4. Is a Polyp Merely a Structural Lesion, or a Manifestation of Profound Uterine Dysbiosis?

For decades, the traditional medical paradigm has regarded endometrial polyps almost exclusively as focal, benign structural anomalies. Their pathogenesis was primarily attributed to excessive tissue proliferation driven by local hormonal imbalances (predominantly hyperestrogenism) or mutations within stromal cells. However, in light of recent research on the uterine microbiome, it has become imperative in gynecology and reproductive medicine to posit a novel, paradigm-shifting pathophysiological hypothesis: in numerous instances, it is hypothesized that the endometrial polyp may not be an isolated anatomical defect, but rather a macroscopic manifestation associated with underlying dysbiosis and chronic uterine inflammation (CE) [13,16,17]. According to this hypothesis, the polyp represents a “tissue reaction” to pathogenic bacterial flora. Under this conceptual framework, mechanical removal alone targets only the structural lesion, rather than the underlying background environment. Unless the homeostasis of the uterine microbiome is restored, the pathogenic environment may still contribute to the development of subsequent lesions [14,18,19].

Therefore, the aim of this narrative review is to critically summarize the current evidence regarding the association between chronic endometritis and endometrial polyps, with particular emphasis on inflammatory and molecular mechanisms, advances in diagnostic strategies, the role of the endometrial microbiome, reproductive implications, and the prognostic significance of CE for polyp recurrence. Finally, we discuss the potential clinical impact of integrating CE assessment into individualized management strategies for women with endometrial polyps.

## 2. Materials and Methods

This narrative review of the literature was performed in accordance with the Scale for the Assessment of Narrative Review Articles (SANRA) recommendations. It explores the current literature regarding chronic endometritis and its relation to endometrial polyps and infertility. A thorough search of electronic databases, including PubMed, Google Scholar, and Scopus, was performed to identify relevant articles regarding the topic. Primarily, articles selected for inclusion were published between January 2010 and July 2026, however some older articles were included from 1996, 2001, 2003, 2005, and 2009 as they contained crucial information regarding certain mechanisms and correlations that could not be found in newer publications. The search strategy used Medical Subject Headings (MeSHs) and free-text terms using Boolean operators using these keywords and combinations: (“chronic endometritis” OR “endometritis” OR “chronic endometrial inflammation”) AND (“endometrial polyps” OR “uterine polyps” OR “intrauterine lesions” OR “infertility” OR “subfertility”). Additionally, manual screening of reference lists from applicable meta-analyses, systematic reviews, and international consensuses was performed to find other potentially relevant papers.

To be eligible for inclusion, studies had to meet the following requirements:

1. Clinical trials, cohort studies, observational studies, international consensuses, systematic reviews, narrative reviews, meta-analyses, and textbooks;
2. Publications regarding chronic endometritis, endometrial polyps and infertility, as well as relevant biochemical pathways related to processes such as inflammation and polyp formation;
3. Articles published in peer-reviewed journals and available in English and Polish.

Articles were excluded based on non-peer-reviewed status, study design (e.g., case reports, conference abstracts, editorials), or a lack of relevance regarding the topic of this paper. Three reviewers (Z.K., T.A., and J.D.) independently conducted the selection process in two stages. At first, titles and abstracts were screened to identify potentially relevant studies. Afterwards, full-text articles were assessed for eligibility. If discrepancies occurred during the selection process, they were discussed among authors seeking input from senior authors (K.C.-W. and M.W.) as needed. Priority was assigned to high-level evidence, including randomized controlled trials, systematic reviews, meta-analyses, large cohort studies, and international consensuses. Data extraction was performed using a standardized collection form according to the SANRA recommendations and included study design, sample size, patient population, diagnostic criteria alongside clinical and biological outcomes. Extracted parameters included diagnostic modalities (hysteroscopy, CD138 immunohistochemistry, histopathology), pathogen and microbiome profiles, inflammatory cytokine markers, reproductive outcomes (e.g., in vitro fertilization [IVF] success, clinical pregnancy rates, live birth rates, recurrent implantation failure, recurrent pregnancy loss), and recurrence rates following treatment. Data were compared across studies to evaluate consistency and divergence across the literature. Synthesis was conducted using a thematic narrative approach, categorizing studies according to their pathophysiological and clinical focus (e.g., diagnostic harmonization, microbial/inflammatory pathways, and therapeutic strategies). Within each category, findings were descriptively appraised to highlight common mechanisms, comparative treatment outcomes, and clinically relevant trends. To improve readability and quality of the English language used, ChatGPT (OpenAI, GPT-5.3, San Francisco, CA, USA) software was used by the authors. Given the narrative design of this review and the methodological heterogeneity of the included literature, a formal meta-analysis and quantitative risk-of-bias assessment were not conducted. Instead, data were synthesized qualitatively around key clinical and biological themes, focusing on diagnostic accuracy, inflammatory mechanisms, treatment response, and subsequent reproductive outcomes.

## 3. Results

The initial literature search in PubMed/MEDLINE yielded 205 records, supplemented by targeted cross-referencing in Scopus and Google Scholar. After removing 12 duplicate entries, 193 records were screened by title and abstract. Of these, 121 were excluded due to irrelevant design, non-peer-reviewed status, or lack of direct focus on chronic endometritis, endometrial polyps, or reproductive failure. Following full-text evaluation of the remaining manuscripts, 72 studies fulfilled all eligibility criteria and were included in the qualitative synthesis. Although the search targeted the literature published between 2010 and 2026, some foundational papers dating from 1996 were also included to provide historical context on diagnostic methods and biochemical pathways. The final evidence base comprised systematic reviews, meta-analyses, prospective and retrospective cohort studies, diagnostic accuracy evaluations, and expert consensus guidelines, originating predominantly from Europe, Asia, and North America.

The included literature was synthesized across three main thematic domains: (1) shared inflammatory, immune, and microbial pathways in chronic endometritis and endometrial polyps; (2) diagnostic concordance between fluid hysteroscopy (e.g., micropolyps, focal hyperemia) and CD138 immunohistochemistry; and (3) the prognostic impact of persistent versus treated chronic endometritis on polyp recurrence and reproductive outcomes (such as IVF success and live birth rates). Overall, the qualitative synthesis demonstrates a consistent clinical and biological association between chronic endometrial inflammation and polyp development, suggesting targeted antibiotic therapy prior to assisted reproduction is a potential strategy warranting rigorous prospective evaluation. Given the narrative design and methodological heterogeneity of the included literature, these findings should be interpreted with consideration of potential publication bias and variable diagnostic criteria across studies.

## 4. Pathophysiology and Molecular Mechanisms

### 4.1. Pathophysiology and Molecular Mechanisms Linking Chronic Endometritis and Endometrial Polyps

The pathogenesis of endometrial polyps (EPs) has traditionally been attributed to local hormonal imbalance, particularly prolonged estrogenic stimulation, altered progesterone responsiveness, and dysregulated cellular proliferation. However, accumulating evidence indicates that these mechanisms alone do not fully explain the development, multiplicity, recurrence, and reproductive consequences of EPs. Instead, current data support a multifactorial model in which chronic endometritis (CE) creates a persistent inflammatory microenvironment that promotes focal endometrial proliferation through complex interactions between immune activation, inflammatory signaling, angiogenesis, extracellular matrix remodeling, hormonal dysregulation, and microbial imbalance [1,10,11,12,13]. Rather than representing independent pathological processes, CE and EPs appear to constitute interconnected manifestations of an altered endometrial ecosystem. Continuous inflammatory stimulation may establish a self-perpetuating cycle in which immune dysregulation promotes structural remodeling of the endometrium, while the resulting polyp further contributes to persistent inflammation and impaired tissue homeostasis [13,14,15,16,17,18,19].

### 4.2. Persistent Inflammatory Activation

On histopathological examination, chronic endometritis is characterized by the presence of plasma cells in the endometrium, most commonly in the stromal layers [20]. Physiological locations of plasma cells include the bone marrow, spleen and Mucosal Associated Lymphoid Tissue (MALT) [21]. Although endometrial mucosa contains a variety of cells involved in immunity, for instance Natural Killer cells, macrophages, T lymphocytes or dendritic cells, healthy endometrium should not contain plasma cells [20,22]. Plasma cells derive from B lymphocytes and produce antibodies in response to inflammation. Most plasma cells are short-lived; thus, if they are detected in an endometrium, it signifies an active inflammatory response [22]. Among the most frequent etiologic factors driving ongoing inflammation of the endometrium are bacteria. Studies identify *Streptococci*, *Enterococcus faecalis* and *Escherichia coli* as the most common agents implicated in CE [20,23]. Additionally, other species including Mycoplasma hominis and *Ureaplasma urealyticum* form the microbiota of an inflamed endometrium [20,23]. A growing body of evidence suggests that viruses, such as *Epstein–Barr Virus* (EBV) or *Human Papilloma Virus* (HPV), can also play a role in CE [24]. Studies debate whether a healthy uterine cavity contains its own microbiota (especially *Lactobacillus* species), and therefore a presence of pathogens signifies endometrial inflammation [25,26]. Upon constant antigenic stimulation by these bacteria and viruses, B lymphocytes produce plasma cells to manage the infection. As chronic endometritis is asymptomatic, in contrast to acute endometritis, its diagnosis is difficult based solely on clinical findings [27]. Patients most often get diagnosed with CE as they struggle with fertility [11,23]. To date, there are no clarified diagnostic criteria for chronic endometritis. Despite the required presence of plasma cells, no specific count is specified to diagnose CE. One useful marker in diagnosis is CD138. It is a surface marker found primarily on mature plasma cells which can be detected via immunohistochemical staining [28]. Despite its specificity to plasma cells, CD138 can sometimes be expressed on various epithelial cells, leading to overdiagnosis of CE [20,29]. According to the Delphi criteria for hysteroscopic diagnosis of CE, it is characterized by the following: (1) strawberry aspects, (2) stromal edema, (3) hemorrhagic spots, (4) focal hyperaemia, and (5) micropolyps [30]. Hysteroscopy offers an alternative, non-invasive pathway to diagnose CE [20]. When compared, histopathological and hysteroscopic findings do not correlate strongly, but a hysteroscopic examination can be a useful additional tool in the diagnosis of CE [31]. Despite the fact that a histopathological examination remains a gold standard for CE diagnosis, more rigorous diagnostic criteria are required to accurately diagnose this condition.

### 4.3. Diagnostics of Chronic Endometritis

**Figure 2:**
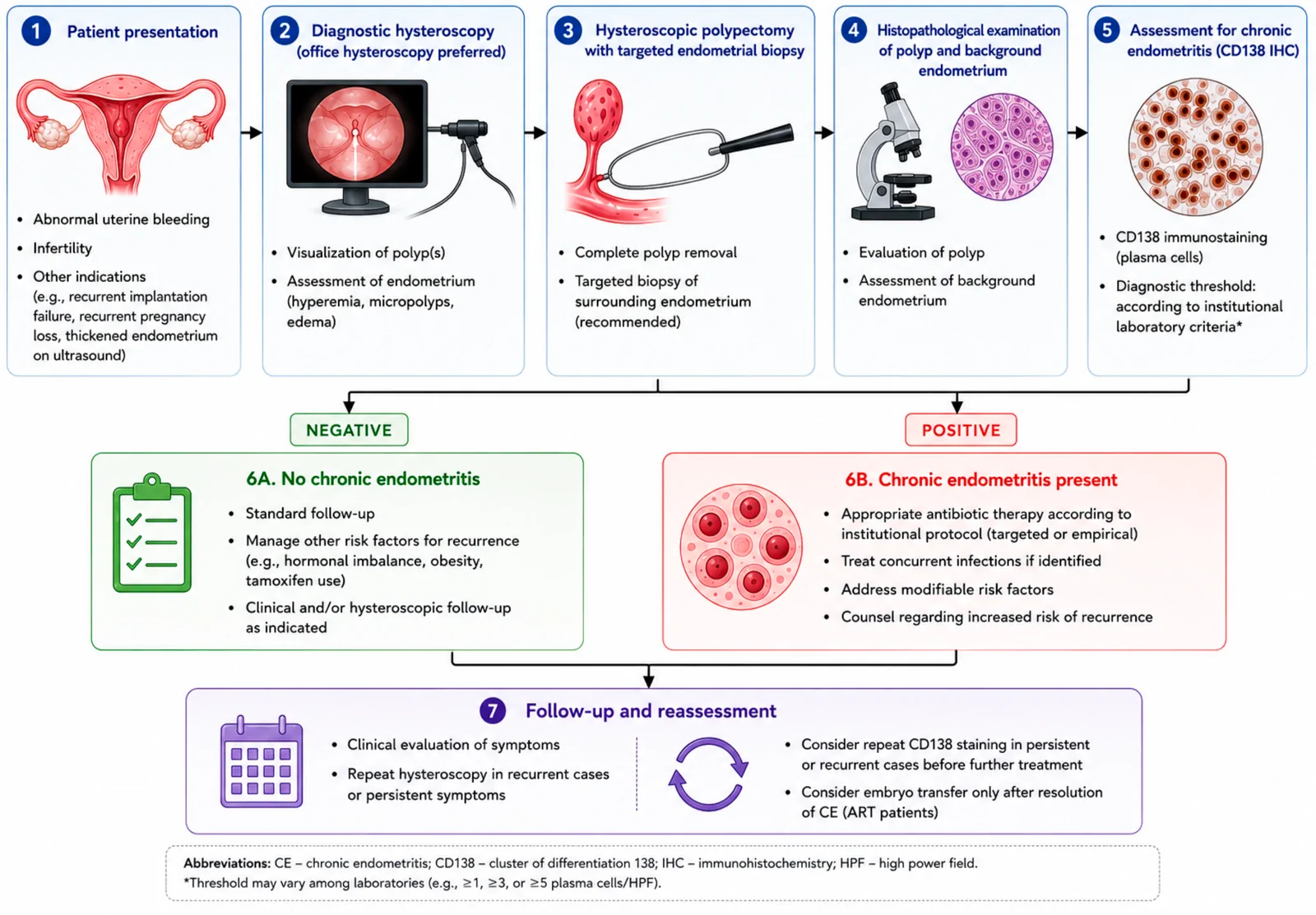
Proposed clinical algorithm for the diagnosis and management of chronic endometritis in women with endometrial polyps.

Hysteroscopy is currently the primary modality for evaluating the uterine cavity in suspected chronic endometritis (CE). A specific diagnostic finding is the presence of micropolyps, defined as endometrial projections measuring less than 2 mm [30]. Rather than simple tissue overgrowths, these structures represent localized aggregations of immune cells associated with underlying inflammation. This pathogenesis is supported by the co-occurrence of the CD138 plasma cell marker, which remains the gold standard for CE diagnosis [15,31,32]. As one of the first studies to describe this issue, the research provides strong evidence for the diagnostic utility of micropolyps in identifying chronic endometritis [17]. These structures were observed in 11.7% (*n* = 96) of the evaluated hysteroscopies, with as many as 93.7% of these cases receiving histopathological confirmation of inflammation [17]. In comparison, among patients without visible micropolyps, the disease was diagnosed significantly less frequently, occurring in only 10.8% of cases (*p* < 0.000001) [17]. The detection of this marker was shown to be associated with a drastic increase in the odds of coexisting disease (OR 124.2; confidence interval: 50.3–205.4) [17]. An evaluation of the test parameters confirmed its high efficacy: specificity was 99%, sensitivity was 54%, while the positive and negative predictive values reached 94% and 89%, respectively [17]. This translated to an overall diagnostic accuracy of 90% [17]. Identifying micropolyps visually yields a specificity of up to 99% for ongoing inflammation, minimizing diagnostic errors [32]. A collaborative study by the Military Institute of Medicine (WIM-PIB) and the Medical University of Warsaw (WUM) demonstrated a highly significant correlation (*p* < 0.0001) between micropolyps and histopathological CE confirmation, reporting a sensitivity of 50% and a specificity exceeding 76% [15]. Similarly, other cohorts have linked micropolyps to confirmed CE in 53.5% of cases (OR 5.27, *p* < 0.001) [33]. Consequently, micropolyps provide the highest positive and negative predictive values among hysteroscopic CE markers [34]. Diagnostic challenges persist in conventional ultrasonography, where physiological mucosal thickening frequently mimics inflammatory edema. During hysteroscopy, reliance on certain subjective visual cues can also lead to misdiagnosis. Studies indicate that focal mucosal hyperemia, pronounced stromal edema, and the “strawberry sign” do not significantly correlate with histological CD138 positivity [15,31]. Therefore, micropolyps remain the most reliable hysteroscopic feature associated with the cellular pathology of CE [15,30,32,33]. Evidence justifying routine screening and treatment for chronic endometritis (CE) has significant limitations (Table 1). Due to a shortage of randomized controlled trials, clinical guidelines do not yet recommend widespread screening [12,15]. This results in a lack of cohesive therapeutic protocols. For example, there is still no consensus on whether antibiotic therapy is necessary following hysteroscopic polypectomy, as the resection of the lesion alone is often sufficient to resolve the inflammation [35]. The key obstacle is the absence of unified diagnostic criteria [12,36]. Although CD138 staining remains the gold standard, researchers use different evaluation methods and varying plasma cell threshold values [36]. Because these cells can occur physiologically in healthy tissue, the adoption of different cutoff points significantly affects the reported prevalence of CE in clinical studies [12,36]. This lack of standardization directly impacts the interpretation of the association between CE and endometrial polyps (EPs) [13]. Although these pathologies frequently coexist, their actual correlation depends strictly on the adopted threshold for the CD138 marker. Studies have shown that only polyps presenting a markedly elevated number of plasma cells demonstrate a significant correlation with the diagnosis of CE [35]. Based on the available evidence, a practical diagnostic and management algorithm can be proposed for women with endometrial polyps and suspected chronic endometritis (Figure 2). The algorithm integrates hysteroscopic evaluation, histopathological examination, CD138 immunohistochemistry, therapeutic decision-making, and follow-up, emphasizing the importance of identifying chronic endometritis to reduce disease recurrence and optimize reproductive outcomes.

### 4.4. Molecular Mechanisms from Chronic Inflammation to Polyp Formation

Chronic endometritis is an inflammatory disease, and thus inflammatory cytokines can be found in endometrial specimens. A study performed by Tortorella et al. revealed that with a 100% sensitivity, women with CE had higher levels of IL-6, IL-1*β*, and TNF-*α* in menstrual effluents compared with those of healthy controls [38]. Upon prolonged TNF-*α* stimulation, there is an increase in estrogen secretion from endometrial glandular cells. Estrogen promotes proliferation of the epithelial cells via IGF-1, EPK/MAPK signaling and various other mechanisms [39]. Excess levels of estrogen can contribute to pathological processes in the uterine cavity, such as abnormal uterine bleeding, endometrial hyperplasia and polyp formation [40]. Chen et al. showed that endometrial polyps exhibit enhanced estrogen signaling compared to a healthy uterine lining based on single-cell RNA sequencing. They also revealed that when in excess, estrogen stimulates stromal cells to signal glandular epithelium to upregulate their growth by WNT, IGF and VEGF pathways [41]. IL-6 also increases estrogen secretion further contributing to endometrial proliferation and polyp formation [42]. Moreover, it acts as an anti-apoptotic factor via various mechanisms, mainly with the JAK/STAT 3 anti-apoptotic proteins, allowing for continuous cell proliferation [42]. IL-1*β* can also contribute to uterine polyp formation, mostly by activating matrix metalloproteinases (MMPs). Upon chronic inflammation, excess IL-1*β* stimulates leukocytes and endometrial stromal cells to produce elevated levels of MMPs, which affects extracellular matrix breakdown and abnormal tissue overgrowth [43]. Köle et al. [44] demonstrated a significantly higher expression of MMP-2 and MMP-9 in endometrial polyp tissues compared to normal endometrium [44]. The same correlation was found by Inagaki et al., where within endometrial polyps, levels of IL-1*β* were associated with higher MMP-2, MMP-9 and overall MMP levels [45]. According to a meta-analysis performed by Vitagliano et al. [13], 51.35% of premenopausal women with endometrial polyps were diagnosed with CE. Additionally, 70.73% of examined polyps had plasma cell infiltration [13]. These findings suggest that chronic inflammation, driven by a bacterial infection, may increase endometrial exposure to inflammatory cytokines, which is hypothesized to stimulate polyp proliferation and angiogenesis and inhibit cell apoptosis within the endometrial polyp. Because both CE and EPs are associated with impaired endometrial receptivity, concurrent management of structural and inflammatory alterations has been proposed to optimize reproductive outcomes, although prospective validation is ongoing [13,46,47,48]. The principal clinical studies investigating the association between chronic endometritis and endometrial polyps are summarized in Table 2, highlighting the evolution of evidence from diagnostic observations to molecular, microbiological, and prognostic investigations.

### 4.5. Recommendations and Applications of Polypectomy

While hysteroscopic polypectomy is widely regarded as the gold standard for the treatment of endometrial polyps [1,5,10], establishing strict clinical indications for surgical intervention remains crucial. According to current guidelines, indications for excision include abnormal uterine bleeding [50,51], infertility [50,51,52], subfertility [50], recurrent implantation failure (RIF), recurrent pregnancy loss [52], and preparation for IVF [50,52]. Furthermore, polypectomy is strictly mandated for postmenopausal women presenting with symptoms or in any scenario where malignancy is suspected [50].

The management of primary asymptomatic polyps in women of reproductive age remains a subject of ongoing debate, often allowing for expectant management [50,51]. The current clinical approach typically shifts when these lesions recur. Excision of recurrent asymptomatic polyps in reproductive-aged women is generally recommended to exclude underlying endometrial pathologies, such as chronic endometritis, and to optimize the endometrial environment for future fertility [52]. Current recommendations from major international scientific societies regarding the diagnosis and management of endometrial polyps are summarized in Table 3.

### 4.6. Is It Worth Considering Antibiotic Therapy in Addition to Polypectomy to Increase IVF Efficacy?

**Figure 3:**
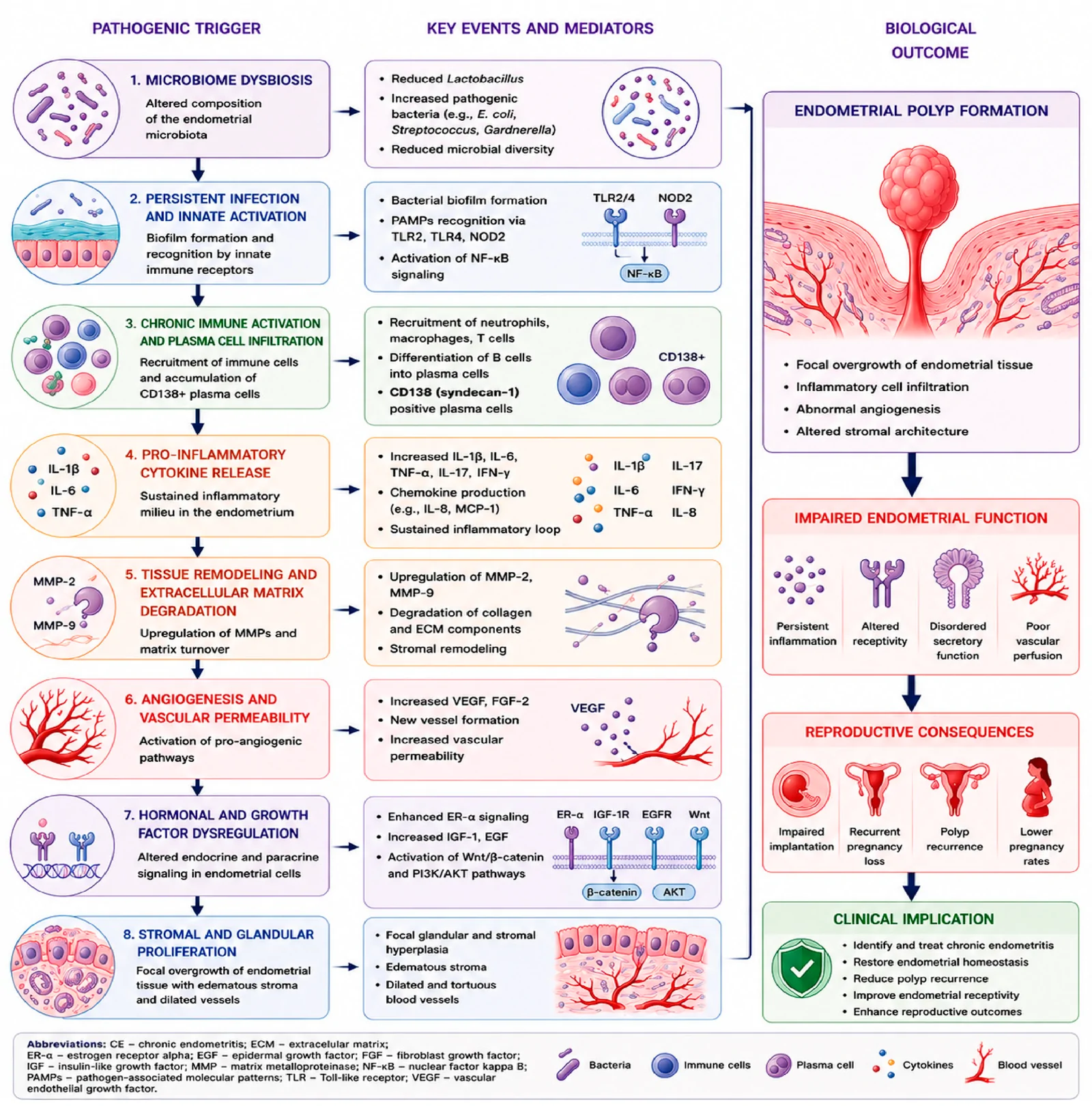
Integrated molecular and cellular pathways linking chronic endometritis with endometrial polyp development and adverse reproductive outcomes. Legend: Chronic endometritis is initiated by alterations in the endometrial microbiota and persistent microbial stimulation, leading to activation of innate and adaptive immune responses with accumulation of CD138-positive plasma cells. Sustained production of pro-inflammatory cytokines (including IL-1β, IL-6, and TNF-α) promotes extracellular matrix remodeling through matrix metalloproteinases, stimulates angiogenesis, and enhances estrogen- and growth factor-dependent signaling pathways. These coordinated molecular events result in focal stromal and glandular proliferation, ultimately leading to endometrial polyp formation. Persistent inflammation further impairs endometrial receptivity and secretory transformation, contributing to recurrent implantation failure, recurrent pregnancy loss, reduced pregnancy rates, and an increased risk of polyp recurrence. Identification and treatment of chronic endometritis may restore endometrial homeostasis, improve reproductive outcomes, and reduce disease recurrence.

Optimizing IVF outcomes in patients diagnosed with endometrial polyps may benefit from a comprehensive approach extending beyond surgical intervention alone, due to the frequent co-occurrence of proliferative lesions with a subclinical inflammatory state. Although hysteroscopic polypectomy effectively eliminates the mechanical barrier hindering embryo implantation [52,53,54], clinical evidence indicates a correlation between the presence of polyps (including micropolyps) and the occurrence of chronic endometritis [17]. CE has been associated with reduced IVF success [47,55,56,57,58], while observational studies suggest that inflammatory resolution following antibiotic therapy [55,59,60,61] correlates with improved pregnancy rates (PRs), live birth rates (LBRs), clinical pregnancy rates (CPRs), and implantation rates (IRs) [55,62,63,64]. For this reason, both polypectomy and antibiotic therapy have been proposed to address both structural and inflammatory factors, potentially supporting endometrial receptivity. Currently available data support a multifactorial model in which chronic endometritis may interact with focal polyp development through a cascade of shared inflammatory, immune, microbial, hormonal, and angiogenic mechanisms. Rather than acting independently, these biological processes are suggested to form a self-perpetuating network that promotes endometrial remodeling, impairs endometrial receptivity, and increases the risk of reproductive failure and polyp recurrence. An integrated overview of these molecular and cellular pathways is presented in Figure 3.

Nevertheless, evidence supporting this multifactorial model is limited and predominantly observational. Several investigations indicate no relationship between treating CE and the improvement of PRs, LBRs, CPRs, and IRs [60,61,63,64,65]. In conclusion, prospective randomized trials are needed to clarify the clinical impact of CE treatment in women with EPs and infertility to establish standardized therapeutic protocols.

## 5. Discussion and Clinical Implications

The present review integrates accumulating evidence indicating that chronic endometritis should no longer be regarded solely as an isolated inflammatory disorder but rather as an integral component of the pathophysiological continuum leading to endometrial polyp formation, recurrence, and impaired reproductive outcomes. Taking into consideration the results of the current scientific research, optimization of the diagnostics and treatment of infertility-related problems calls for the update of the existing standards. On the basis of the conducted literature review, the following conclusions for clinical practice can be made.

### 5.1. The Need for Additional Histopathological Investigation of Endometrial Polyps

The coexistence of endometrial polyps and chronic endometritis is a relatively common condition, which is frequently asymptomatic or oligosymptomatic, but greatly affects the receptivity of the endometrium [66]. Traditional histopathological analysis based only on hematoxylin and eosin (H&E) staining may underdiagnose subclinical stromal inflammation [37]. Hence, in case of polyp removal, in particular among women who experience infertility or recurrent implantation failure, additional histopathological investigation, such as immunohistochemistry with CD138 plasma cells, could be a novel integrated and valuable strategy [67,68,69]. Such an approach eliminates a critical diagnostic gap and enables identification of the underlying inflammatory etiology of the infertility problem, serving as a part of individualized treatment plans.

### 5.2. Therapeutic Strategies for Endometrial Inflammation and Dysbiosis Before ART

When chronic endometritis is identified prior to embryo transfer, targeted or broad-spectrum antibiotic therapy is frequently proposed to restore the physiological state of the immunological microenvironment in the uterine cavity [68]. Preliminary findings from meta-analyses [63,69] and clinical studies [68] suggest that elimination of CE via targeted pharmacotherapy might improve implantation, clinical pregnancy, and live births in IVF/ICSI procedures. This is particularly relevant for patients dealing with recurrent implantation failure [63,68]. Thus, resolution of chronic endometrial inflammation before subsequent ART becomes a stage of clinical treatment worth considering [63,69].

An additional potential therapeutic approach includes the use of probiotics in women with CE. Hu et al. examined combining oral doxycycline with vaginal *Lactobacillus*. Although patients on combined antibiotics with probiotics exhibited higher rates of biochemical pregnancy compared to patients on doxycycline alone, the results were not statistically significant [70]. Studies on mice presented beneficial results of *Lactobacillus crispatus* on reducing endometrial inflammation and enhancing embryo implantation, which poses as a promising intervention to improve pregnancy rates in CE patients [71]. In a study by Kyono et al., oral probiotic administration in patients with a non-*Lactobacillus*-dominated endometrial microbiome successfully shifted the profile toward *Lactobacillus* dominance; however, this did not translate into statistically significant differences in pregnancy rates compared to those with persistent dysbiosis [72]. Despite these potential positive reproductive outcomes, strict recommendations regarding routine targeted antibiotic or probiotic therapy before ART are currently lacking due to limited high-level evidence regarding their true impact on reproductive success.

## 6. Future Perspectives

Despite substantial advances in understanding the relationship between CE and EPs, several fundamental questions remain unresolved. The greatest limitation of the current evidence is the predominance of retrospective and observational studies, which preclude definitive conclusions regarding causality. Consequently, whether chronic inflammation initiates polyp development or represents a secondary response to an existing lesion remains uncertain.

Standardization of CE diagnosis should be considered a research priority. Considerable variability persists regarding the optimal threshold for CD138-positive plasma cells, biopsy timing within the menstrual cycle, immunohistochemical protocols, and interpretation of hysteroscopic findings. Establishing internationally accepted diagnostic criteria would substantially improve comparability across future studies and facilitate evidence-based clinical recommendations. Artificial intelligence-assisted hysteroscopy, computer-assisted CD138 quantification, radiomics, and microbiome-based predictive models may substantially improve personalized diagnosis and management of CE in the coming years.

Future investigations should also focus on integrating molecular and translational approaches into the study of endometrial pathology. Advances in single-cell transcriptomics, spatial transcriptomics, proteomics, metabolomics, and next-generation sequencing provide unprecedented opportunities to characterize the inflammatory and microbial landscape of the endometrium. These technologies may identify molecular signatures associated with recurrent polyps, reproductive failure, or response to treatment, enabling more precise patient stratification.

Particular attention should be directed toward the role of the endometrial microbiome. Although microbial dysbiosis has emerged as a promising contributor to chronic inflammation, its causal relationship with EP formation remains incompletely understood. Prospective longitudinal studies evaluating microbiome dynamics before and after hysteroscopic polypectomy, with or without targeted treatment of CE, are needed to determine whether restoration of microbial homeostasis influences recurrence rates or reproductive outcomes.

Artificial intelligence and digital pathology may also transform the diagnosis of CE. Machine-learning algorithms capable of identifying plasma cells or recognizing subtle hysteroscopic inflammatory patterns could improve diagnostic reproducibility while reducing interobserver variability. Such technologies may ultimately support more standardized and objective assessment of chronic endometrial inflammation.

Finally, well-designed randomized controlled trials are required to determine whether routine evaluation and treatment of CE improve clinically meaningful outcomes, including live birth rates, implantation success, recurrence-free survival after polypectomy, and patient-reported quality of life. These studies will be essential before incorporating CE assessment into routine management algorithms for women with endometrial polyps.

## 7. Limitations

The main limitation of this study is the heterogeneity of included studies. Additionally, restriction to English-language and Polish-language publications may have caused the exclusion of relevant international reports and implement a language bias. As a narrative review, there was no quantitative meta-analysis, which increases the risk of publication bias and limits the ability to directly imply the statistical significance of its findings. Findings of this study should be interpreted with appropriate caution due to no formal risk-of-bias assessment. Furthermore, substantial heterogeneity exists among studies regarding diagnostic thresholds for CD138-positive plasma cells and definitions of chronic endometritis, limiting direct comparison across studies.

## 8. Conclusions

Accumulating evidence indicates that chronic endometritis represents an important component of the biological environment associated with endometrial polyp development and recurrence. Rather than viewing endometrial polyps solely as localized hormone-dependent proliferative lesions, contemporary evidence supports a broader concept in which persistent inflammation, immune dysregulation, microbial alterations, abnormal angiogenesis, extracellular matrix remodeling, and hormonal signaling interact to shape an inflammatory endometrial phenotype.

Although a causal relationship has not yet been conclusively established, chronic endometritis appears to be consistently associated with endometrial polyps, particularly in women with recurrent lesions and infertility. This evolving understanding provides a plausible biological explanation for the persistence and recurrence of disease despite technically successful hysteroscopic treatment and highlights the importance of considering the surrounding endometrium in addition to the polyp itself.

At present, routine screening and treatment of chronic endometritis cannot be recommended for all women with endometrial polyps because of heterogeneous diagnostic criteria and limited prospective evidence. However, evaluation for CE may be appropriate in carefully selected patients, particularly those with recurrent polyps, repeated implantation failure, recurrent pregnancy loss, or otherwise unexplained infertility.

Future progress will depend on the standardization of diagnostic criteria, validation of molecular and microbiome-based biomarkers, and high-quality prospective clinical trials. A more comprehensive understanding of the inflammatory mechanisms underlying endometrial pathology may facilitate the transition from purely lesion-oriented treatment toward individualized management strategies that address the biological processes responsible for disease development, recurrence, and impaired reproductive function.

## Figures and Tables

**Table 1:** Diagnostic challenges and variability in chronic endometritis identification: clinical and interpretive implications.

Diagnostic Limitation	Description of the Problem in Clinical Practice
Lack of a unified plasma cell cutoff threshold	Different centers adopt values (e.g., ≥1 to ≥5 plasma cells per high-power field [HPF]) as the diagnostic standard, precluding standardization [31].
Cross-reactivity (false-positive results)	CD138 antibodies can stain endometrial glandular epithelial cells, fibroblasts, and B lymphocytes [31], which may erroneously inflate plasma cell counts and lead to overdiagnosis [20,31,37].
Blind biopsy limitations (false-negative results)	Cellular infiltration in CE is often focal. A blind biopsy (e.g., Pipelle) may fail to sample a representative area, resulting in a missed diagnosis, which further confounds treatment outcomes [37].
Physiological presence of plasma cells	Plasma cells can be found in the endometrium of women with, e.g., abnormal uterine bleeding without any association with an active inflammatory process [31].

**Table 2:** Landmark clinical studies investigating the association between chronic endometritis and endometrial polyps.

Study	Year	Study Design	Population	Principal Findings	Clinical Implications
**Cicinelli et al.** [17]	2005	Prospective diagnostic study	Women undergoing office hysteroscopy	Endometrial micropolyps were highly associated with histologically confirmed CE (93.7%); specificity 99%, PPV 94%.	Micropolyps represent the most specific hysteroscopic marker of CE and should prompt endometrial biopsy with CD138 immunohistochemistry.
**Cicinelli et al.** [23]	2008	Prospective observational study	2190 women undergoing office hysteroscopy	Demonstrated significant correlations between hysteroscopic, histopathological and microbiological findings in CE.	Supports combined hysteroscopic and histopathological evaluation rather than relying on a single diagnostic modality.
**Vitagliano et al.** [13]	2021	Systematic review and meta-analysis	Eight observational studies	Approximately 51% of premenopausal women with endometrial polyps had CE; plasma cell infiltration was identified in approximately 71% of polyps.	Strongest evidence supporting an inflammatory component in endometrial polyp pathogenesis.
**Nomiyama et al.** [35]	2021	Retrospective cohort	Infertile women undergoing hysteroscopic polypectomy	Polyps containing increased plasma cell infiltration were strongly associated with CE in the surrounding endometrium.	Histological evaluation should include both the polyp and adjacent endometrium, particularly in infertility patients.
**Liang et al.** [49]	2023	Cross-sectional microbiome study	Infertile women with CE and/or EPs	Altered genital tract microbiota was observed in women with CE and endometrial polyps, suggesting shared microbial characteristics.	Supports the hypothesis that dysbiosis contributes to chronic inflammation and polyp formation.
**Qu et al.** [18]	2023	Retrospective cohort	Premenopausal women after hysteroscopic polypectomy	CE independently increased the risk of endometrial polyp recurrence after surgery.	Identification and treatment of CE may reduce recurrence following hysteroscopic polypectomy.
**Vaduva et al.** [14]	2023	Observational study	Women undergoing IVF evaluation	CE occurred significantly more frequently in women with endometrial polyps and was associated with poorer reproductive outcomes.	Women with infertility and polyps should be evaluated for concomitant CE before Assisted Reproductive Technology (ART).
**Zhao et al.** [16]	2024	Case–control microbiome study	Women with endometrial polyps and controls	Women with polyps exhibited altered endometrial microbiota composition compared with healthy controls.	Provides molecular evidence supporting the role of microbial dysbiosis in EP development.
**Szafarowska et al.** [15]	2025	Diagnostic cohort	Women undergoing hysteroscopy with CD138 assessment	Micropolyps showed the strongest correlation with CD138-positive CE among hysteroscopic findings.	Reinforces the diagnostic value of micropolyps as a marker warranting histological confirmation.
**Kitaya et al.** [20]	2026	International expert consensus (modified Delphi)	International multidisciplinary expert panel	Proposed standardized terminology and diagnostic recommendations for CE, including the role of hysteroscopy and CD138 immunohistochemistry.	Establishes an international framework for future clinical practice and research on CE.

**Table 3:** Current recommendations of major international scientific societies regarding the diagnosis and management of endometrial polyps.

Clinical Scenario	AAGL [50]	SOGC [51]	British Fertility Society [52]
Asymptomatic polyp <10 mm	Consider observation	Observation acceptable	Individualized
Abnormal uterine bleeding	Polypectomy recommended	Polypectomy recommended	Individualized
Infertility	Polypectomy recommended	Polypectomy recommended	Polypectomy recommended
Subfertility	Polypectomy recommended	No recommendations	Consider co-existence of another endometrial pathology
Before IVF	Polypectomy recommended	Consider polypectomy	Polypectomy recommended
Recurrent implantation failure	No recommendations	No recommendations	Polypectomy recommended
Recurrent pregnancy loss	No recommendations	Consider polypectomy	Polypectomy recommended
Asymptomatic Postmenopausal polyp	Consider observation	Consider polypectomy	No recommendations
Symptomatic postmenopausal polyp	Polypectomy recommended to exclude malignancy	Consider polypectomy	No recommendations
Suspicion of malignancy	Polypectomy recommended	Hysteroscopy recommended	Hysteroscopy recommended
Recurrent polyps	Polypectomy via direct hysteroscopic visualization to reduce recurrence rates	Polypectomy via direct hysteroscopic visualization to reduce recurrence rates	No recommendations
Symptomatic premenopausal polyp	Polypectomy recommended	Consider referral to a gynecologist	No recommendations
Asymptomatic polyp in low-risk patients	Expectant management	Expectant management	No recommendations

## Data Availability

The original contributions presented in this study are included in the article. Further inquiries can be directed to the corresponding authors.

## Abbreviations

The following abbreviations are used in this manuscript:

CE	chronic endometritis
EP	endometrial polyp
IVF	in vitro fertilization
PR	pregnancy rate
LBR	live birth rate
CPR	clinical pregnancy rate
IR	implantation rate
EBV	Epstein–Barr Virus
HPV	Human Papilloma Virus
